# pyPheWAS Explorer: a visualization tool for exploratory analysis of phenome-disease associations

**DOI:** 10.1093/jamiaopen/ooad018

**Published:** 2023-04-03

**Authors:** Cailey I Kerley, Tin Q Nguyen, Karthik Ramadass, Laurie E Cutting, Bennett A Landman, Matthew Berger

**Affiliations:** Department of Electrical & Computer Engineering, Vanderbilt University, Nashville, Tennessee, USA; Vanderbilt Brain Institute, Vanderbilt University Medical Center, Nashville, Tennessee, USA; Department of Special Education, Peabody College of Education and Human Development, Nashville, Tennessee, USA; Department of Computer Science, Vanderbilt University, Nashville, Tennessee, USA; Vanderbilt Brain Institute, Vanderbilt University Medical Center, Nashville, Tennessee, USA; Department of Special Education, Peabody College of Education and Human Development, Nashville, Tennessee, USA; Department of Electrical & Computer Engineering, Vanderbilt University, Nashville, Tennessee, USA; Vanderbilt Brain Institute, Vanderbilt University Medical Center, Nashville, Tennessee, USA; Department of Computer Science, Vanderbilt University, Nashville, Tennessee, USA; Vanderbilt University Institute of Imaging Science, Vanderbilt University, Nashville, Tennessee, USA; Department of Biomedical Engineering, Vanderbilt University, Nashville, Tennessee, USA; Department of Computer Science, Vanderbilt University, Nashville, Tennessee, USA

**Keywords:** electronic health records, PheWAS, ICD codes, interactive visualization

## Abstract

**Objective:**

To enable interactive visualization of phenome-wide association studies (PheWAS) on electronic health records (EHR).

**Materials and Methods:**

Current PheWAS technologies require familiarity with command-line interfaces and lack end-to-end data visualizations. pyPheWAS Explorer allows users to examine group variables, test assumptions, design PheWAS models, and evaluate results in a streamlined graphical interface.

**Results:**

A cohort of attention deficit hyperactivity disorder (ADHD) subjects and matched non-ADHD controls is examined. pyPheWAS Explorer is used to build a PheWAS model including sex and deprivation index as covariates, and the Explorer’s result visualization for this model reveals known ADHD comorbidities.

**Discussion:**

pyPheWAS Explorer may be used to rapidly investigate potentially novel EHR associations. Broader applications include deployment for clinical experts and preliminary exploration tools for institutional EHR repositories.

**Conclusion:**

pyPheWAS Explorer provides a seamless graphical interface for designing, executing, and analyzing PheWAS experiments, emphasizing exploratory analysis of regression types and covariate selection.

## INTRODUCTION

The past few decades have seen a surge in the availability of electronic health record (EHR) data^1^ and, unsurprisingly, numerous methods for making sense of this rich data source.^2^^,^^3^ Phenome-wide association studies (PheWAS) are often used to identify associations between a genotype and many EHR phenotypes, often derived from International Classification of Disease (ICD) billing codes.^4^ This technique has discovered novel associations between EHR phenotypes and HLA-DRB1*1501^5^ and determined the contribution of Neanderthal genetic variants to phenotypes of modern humans.^6^ Since its introduction, the PheWAS method has expanded to nongenetic applications as well; such studies include a characterization of co-occurring phenotypes in autism spectrum disorder^7^ and a scan for phenotype associations with white blood cell count in an intensive care unit cohort.^8^

Currently, several tools exist for running PheWAS experiments and related enrichment analyses (Table 1). Many of these tools obscure data flow, requiring the user to navigate several command-line interfaces or write their own code; others have engaging graphical interfaces but only provide visualizations for the final output. All of these existing tools neglect the difficult task of assessing and visualizing model inputs. These factors can make verification of model designs difficult and present unnecessary barriers for researchers, especially those who are experimenting with PheWAS for the first time. To bridge this accessibility gap, we present pyPheWAS Explorer, an interactive visualization tool for the analysis of ICD-derived phenotypes. Inspired by RegressionExplorer,^9^ pyPheWAS Explorer provides detailed inspection of model inputs, real-time model building, and multifaceted result visualization.

**Table 1.** Comparison of pyPheWAS Explorer with existing PheWAS tools

	R PheWAS^10^	pyPheWAS^11^	PHESANT^12^	PheWAS-ME^13^	pyPheWAS Explorer
Dataset agnostic	X	X		X	X
Interactive graphical user interface			X	X	X
PheWAS core analysis	X	X	X		X
PheWAS input visualization					X
PheWAS output visualization	X	X	X	X	X
Post-PheWAS enrichment analysis		X		X	

## MATERIALS AND METHODS

**Figure 1.**
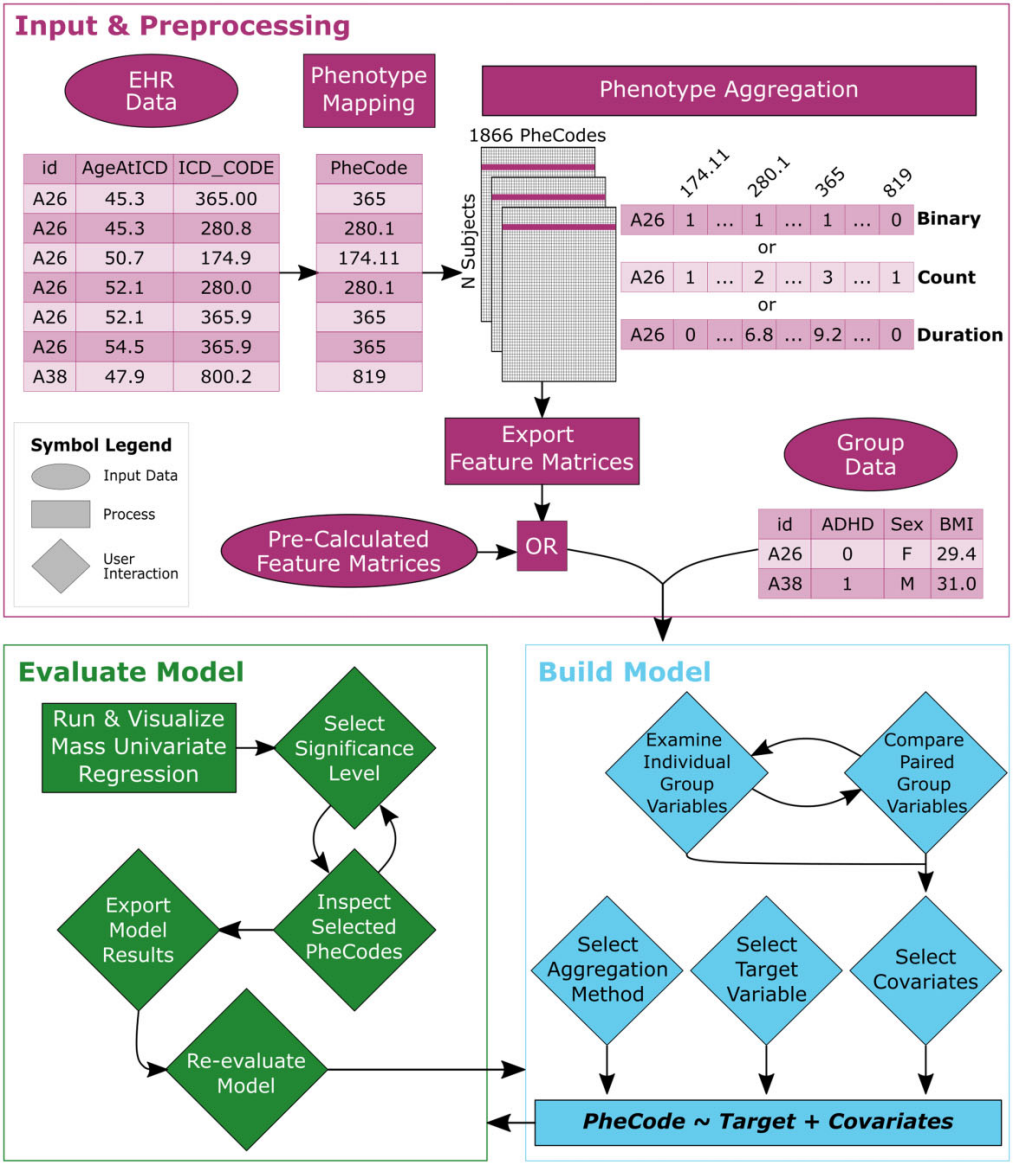
pyPheWAS Explorer workflow. All data preprocessing is done automatically in the background; feature matrices are saved for faster startup in subsequent sessions. In the model building phase, the user may examine group variables and compare them to each other before choosing the PheWAS model target variable and covariates. Additionally, users may specify the type of PheCode aggregation (binary, count, or duration). In the model evaluation phase, the user examines mass univariate regression results at configurable significance levels. Based on these results, the user may move back into the model building phase to re-evaluate their model design.

The pyPheWAS Explorer workflow (Figure 1) is composed of 3 phases: input and preprocessing, model building, and model evaluation. In the following sections, we describe each of these phases in detail, after briefly outlining PheWAS experiments in general.

### A brief description of PheWAS

A PheWAS aims to identify associations between a single variable of interest (here referred to as the target variable) and many EHR-derived phenotypes. This target may be any genetic (eg, single nucleotide polymorphisms) or nongenetic (e.g. disease diagnosis) binary variable. Briefly, phenotype associations are found as follows. (1) EHR data are mapped to phenotypes and (2) these phenotypes are aggregated across each patient’s record. Importantly, this mapping and aggregation process reduces the complexity inherent in EHR coding, thereby narrowing the phenome to more meaningful phenotypes and increasing statistical power. Next, (3) mass univariate regression is performed on each phenotype and (4) the regression results are visualized for interpretation. For more depth, the PheWAS method is described in full elsewhere.^11^^,^^14^^,^^15^

pyPheWAS Explorer performs steps 1 and 2 of this procedure in the background during the input and preprocessing phase, after which the user may interactively build and run the PheWAS regression model (step 3). Finally, the user may interpret target-phenotype associations by directly interacting with both tabular and visual representations of the PheWAS model results (step 4).

### Input and preprocessing

The first phase of the Explorer workflow involves transforming the longitudinal EHR into phenotype feature matrices (Figure 1). Two data files are required: a group demographics file and an EHR file (examples may be found online at https://github.com/MASILab/pyPheWAS). The group file contains potential target variables along with other demographic information (eg, sex and body mass index) for all patients in the cohort. The EHR file contains ICD records, where each record consists of the subject’s unique identifier, an ICD-9-CM or ICD-10-CM code, and the subject’s age at the time of the recorded event. pyPheWAS Explorer first maps all of these ICD codes to a set of 1866 phenotype codes (PheCodes).^16^^,^^17^ It is important to note that though these tables are extensive, they are incomplete; any ICD codes not included in the mapping are removed from the study.

The Explorer then uses 3 different aggregation methods to summarize the existence of each PheCode across each patient’s EHR. *Binary* aggregation considers the relationship between the target variable and the presence of each PheCode; this feature matrix contains only zeros (the PheCode was absent in the patient’s record) and ones (the PheCode was present in the patient’s record). *Count* aggregation considers the relationship between the target variable and the number of occurrences of each PheCode; this feature matrix contains positive integers corresponding to the total number of instances of each PheCode in each patient’s record. *Duration* aggregation considers the relationship between the target variable and the span of time over which a PheCode was present; this feature matrix contains the time in years between the first and last occurrences of each PheCode in each patient’s record.

### Building a PheWAS model

**Figure 2.**
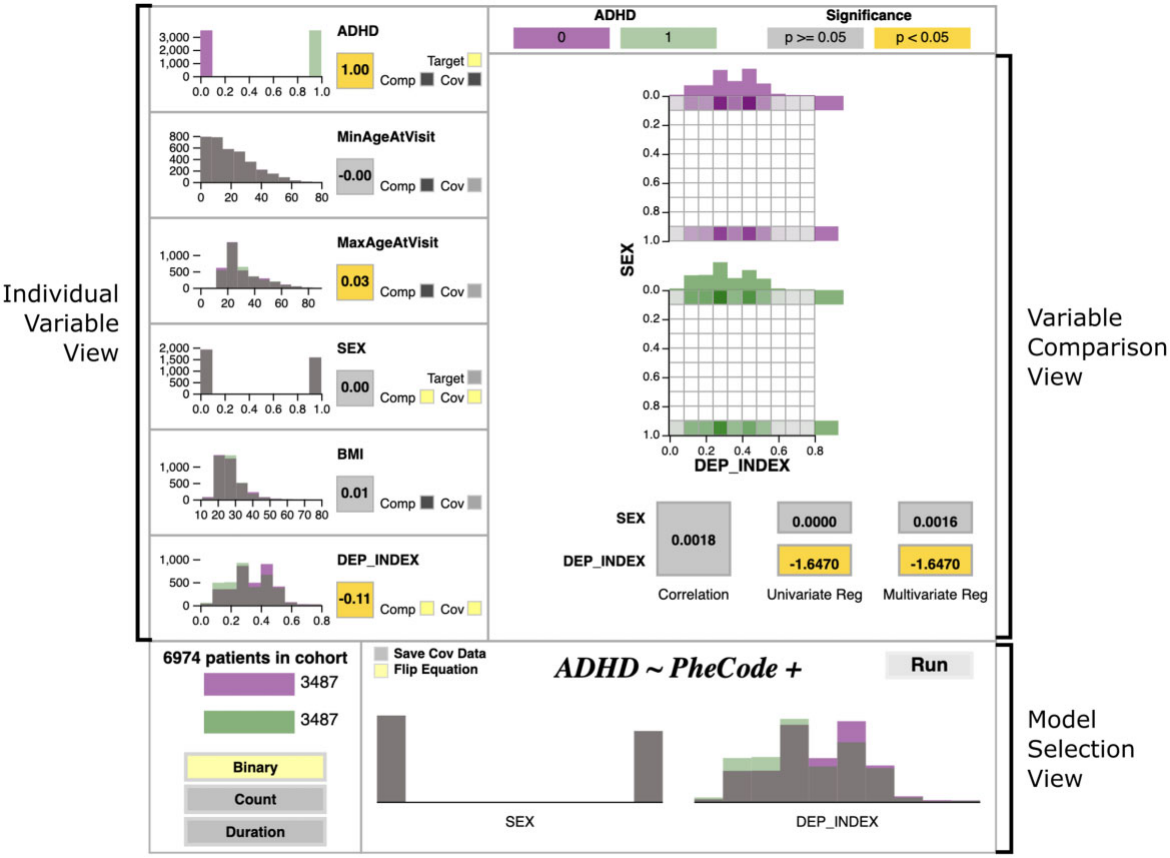
pyPheWAS Explorer Regression Builder Panel. For demonstration, a cohort of ADHD cases and non-ADHD controls is shown. Group variables in this dataset included minimum/maximum age at visit (MinAgeAtVisit/MaxAgeAtVisit), biological sex, body mass index (BMI), and deprivation index (DEP_INDEX). The right side of this panel shows the variables sex and deprivation index loaded into the variable comparison view, while the model selection view shows the same variables added to a binary PheWAS model. Color encodings for the case and control groups, correlations, and regression coefficients are shown along the top bar.

Model building in pyPheWAS Explorer is performed via the interactive panel (Figure 2). The individual variable view facilitates informed PheWAS target variable and covariate selection by enabling detailed inspection of each variable γ in the group file. This view presents the correlation coefficient between γ and the target variable (represented by a colored block) and a histogram of γ values (separated by target group). The overlapping histogram allows the user to check case/control matching for each potential covariate, while the correlation coefficient allows the user to identify potential covariate biases. If γ is binary, the *Target* button in this view allows the user to designate γ as the PheWAS target variable (initially, the target variable is chosen arbitrarily from the available binary variables in the group file). For all nontarget variables, the *Cov* button allows the user to add γ to the PheWAS model as a covariate.

Typically, PheWAS covariates are chosen based on clinical significance, interesting distribution patterns, or user expertise. When considering the inclusion of multiple covariates, the user may first wish to examine intercovariate relationships, particularly when they suspect that the chosen variables may violate the regression model’s independence assumption. For this purpose, the Explorer’s variable comparison view (Figure 2) allows the user to compare pairs of variables chosen via the *Comp* button in the individual variable panels. For a qualitative independence assessment, the joint distribution of these variables is provided, again separated by target group; this includes a grid that captures the overlap of selected variable distributions, along with the individual histograms for each variable at the top and right of the grid. Hovering over the joint distribution grid allows the user to query the value of each bin. A quantitative assessment is also provided via 2 statistical tests. The first of these is the correlation coefficient between the selected variables. The second is a multicollinearity test, wherein the target variable is regressed as a function of each variable individually and by both variables together. The coefficients calculated from the correlation and regressions are all overlaid on colored blocks, where the color indicates statistical significance. In general, if the 2 variables are not correlated and their regression coefficients remain constant across the individual and combined multicollinearity models, the independence assumption holds, and they may be safely included in the PheWAS model together.^18^

Finally, the model selection view allows users to build the PheWAS model. Here, a list of buttons allows the user to select a PheCode aggregation type (binary, count, or duration). Additionally, the user may choose to make the target variable either the dependent variable or an independent predictor in the regression equation. Logistic regression^19^ is used for models with a binary dependent variable, while linear regression^20^ is used for models with nonbinary dependent variables (eg, count or duration PheCode aggregates).

### Evaluating a PheWAS model

**Figure 3.**
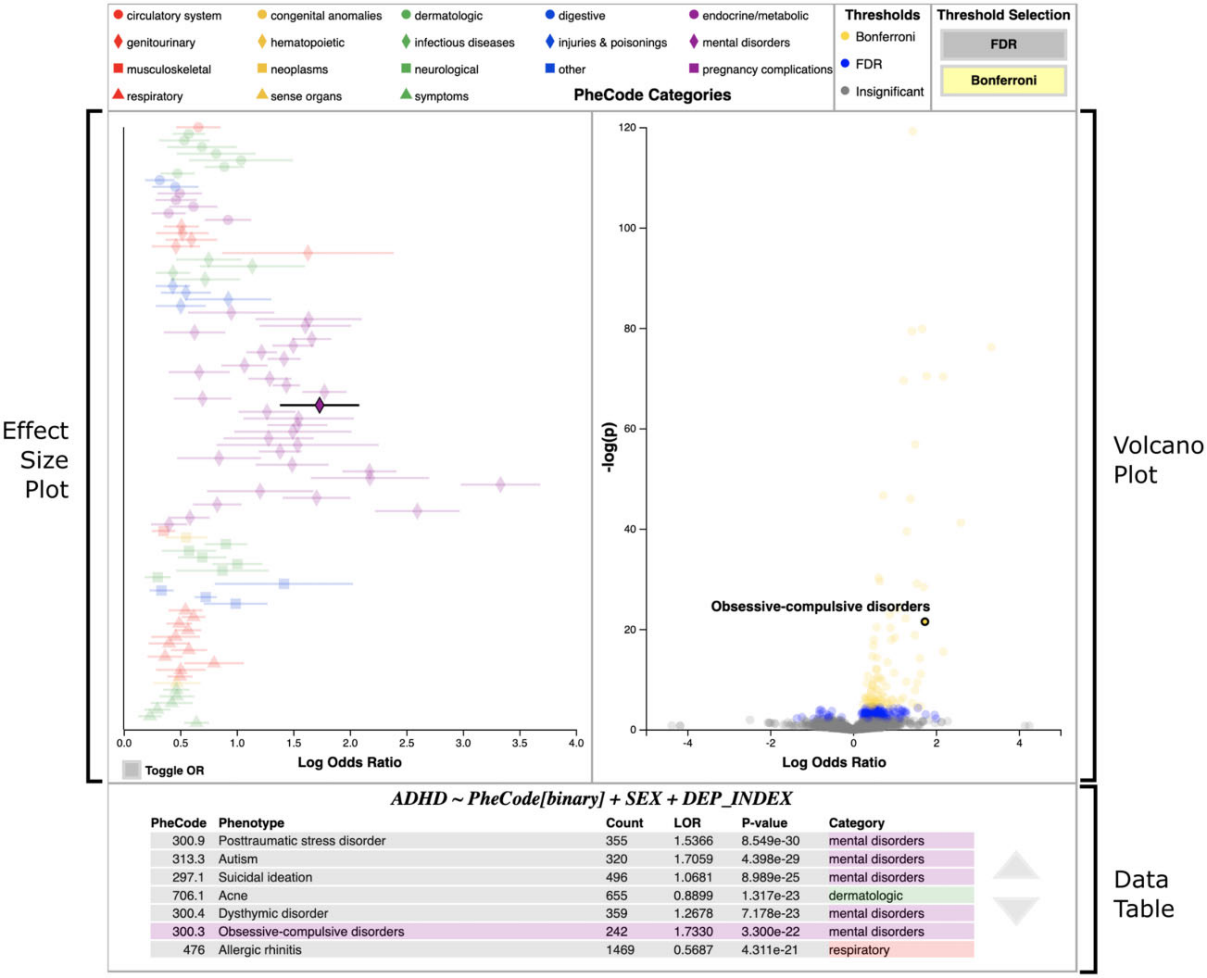
pyPheWAS Explorer Regression Evaluation Panel. PheWAS results from the binary ADHD model are shown in 3 linked views: an effect size plot, volcano plot, and data table. Selecting a PheCode in any view highlights it in the other 2 views; PheCode 300.3, Obsessive-compulsive disorders, is selected for demonstration. The significance threshold for the effect size plot may be toggled between FDR and Bonferroni multiple comparisons correction by selecting the corresponding buttons at the top of the panel; here, Bonferroni is applied. Color legends for the effect size plot (PheCode categories) and volcano plot (significance thresholds) are shown along the top bar.

Selecting the *Run* button in the model building panel triggers a real-time estimation of the user’s model; the results of this estimation are automatically displayed on the evaluation panel in 3 linked views (Figure 3). Selecting a data point in any of these views highlights the corresponding data point in the other 2 views.

The volcano plot presents an overview of the entire experiment. PheCode-target association effect size is shown on the x-axis with significance shown on the y-axis. Marker color in this plot corresponds to which multiple comparisons correction threshold a PheCode exceeds (Bonferroni,^21^ False Discovery Rate (FDR),^22^ or insignificant). This view serves as a starting point for deeper investigations, as users can see interesting PheCode relationships in a single glance.

The effect size plot presents the PheCode-target association effect size (with confidence interval) on the x-axis, with PheCodes listed down the y-axis. Only PheCodes that exceed the user-selected multiple comparisons correction significance threshold (either FDR^22^ or Bonferroni^21^) are included in this plot. Marker color and shape in the effect size plot correspond to 18 descriptive PheCode categories.

Finally, the data table provides the most detailed view, listing each PheCode’s category, regression coefficient, *P*-value, and the number of subjects that have at least 1 record of that PheCode. These tabular results are sorted so that the most significant results are at the top of the table. This data table is automatically saved so that users may reference it after closing pyPheWAS Explorer.

### Installation and use

pyPheWAS Explorer is available in open source as part of the pyPheWAS Python package^11^ (version 4.1+), available at https://github.com/MASILab/pyPheWAS. The Explorer consists of a JavaScript and D3^23^ front-end, with a Python Flask^24^ server back-end. A tutorial video detailing installation and usage is included as Supplementary material with this article.

### Software evaluation

To evaluate the Explorer software, we conduct an exploratory analysis of attention deficit hyperactivity disorder (ADHD) subjects compared to matched controls. A deidentified EHR dataset was acquired from the Synthetic Derivative at Vanderbilt University Medical Center.^25^ A total of 3487 ADHD subjects were identified as those with at least 3 records of ICD-9-CM code 314.01 or ICD-10-CM codes F90, F90.0, F90.1, F90.2, F90.8, or F90.9. ADHD subjects were matched one-to-one with non-ADHD controls based on biological sex and minimum age at visit (±0.1 years). Performance benchmarks for this dataset and a synthetic dataset (used in the Supplementary tutorial video) are shown in Table 2; these benchmarks were performed on a 2.6 GHz Quad-Core Intel i7 with 16 GB of memory.

**Table 2.** Explorer runtime performance (seconds) for ADHD and synthetic cohorts

	ADHD cohort	Synthetic cohort
No. of patients	6 974	10 000
No. of ICD records	1 014 832	103 493
Startup (build feature matrices) (s)	56.3	27.6
Startup (load feature matrices) (s)	5.3	7.6
Variable comparison processing (s)	0.4	0.4
PheWAS regression processing (binary) (s)	105.0	10.6
PheWAS regression processing (duration) (s)	34.9	9.0

## RESULTS

Figure 2 shows the ADHD cohort loaded into pyPheWAS Explorer’s regression builder interface, with *ADHD* chosen as the target variable. From the individual variable views, we confirm that the case and control groups were matched on sex and minimum visit age due to their histograms’ perfect overlap, while the other group variables show diverging distributions. Additionally, we see that most variables are uncorrelated with ADHD, but interestingly, deprivation index, a measure of socioeconomic status where a higher value corresponds to higher deprivation,^26^ has a slightly negative correlation (ie, ADHD subjects tend to be less deprived than controls). Based on this, we are interested in building a PheWAS model that includes both deprivation index and sex as covariates. To ensure that these covariates may be used together safely, we first examine them using the variable comparison view. The 2 variables are not highly correlated, and their regression coefficients remain constant across the individual and combined multicollinearity tests; so, we conclude that they are sufficiently independent and add them to our model. Finally, we select the *binary* model type and compute our PheWAS model.

We use the volcano plot (Figure 3, Supplementary Figure S1) to examine PheCode associations with the highest combined effect and significance; these include anxiety disorder, mood disorders, pervasive developmental disorders, and major depressive disorder, all of which are known to co-occur with ADHD.^27–29^ We select Bonferroni correction for the effect size plot and find that all significant results have positive effects, with many in the “mental disorders” category. More interesting, however, are the less prominent PheCode categories (Supplementary Table S1): “dermatologic” (acne; rash and other nonspecific skin eruption), “endocrine/metabolic” (abnormal weight gain), and “respiratory” (bronchitis; acute sinusitis). To investigate these further, we change the PheCode aggregation type to *duration* and recompute our PheWAS model (Supplementary Figure S2); each of these “interesting” PheCodes remains in the effect size plot. This re-evaluation suggests that these 3 PheCode categories may be candidates for deeper study, as they presented significant effects in both EHR presence and duration and are potentially less known to be associated with ADHD.

## DISCUSSION

pyPheWAS Explorer is a straightforward visual interface that captures a comprehensive summary of the entire PheWAS experiment, making rapid prototyping and interpretation of PheWAS models possible. The Explorer does currently have several limitations. Preparing EHR for the Explorer is not trivial. The full pyPheWAS package contains some data preparation tools, but these require familiarity with a command line, as does installing and launching pyPheWAS Explorer. Extending an analysis to a new dataset requires reloading and repeating the original steps in the Explorer interface, which may harm reproducibility efforts. Additionally, though the presented ADHD use case demonstrates the functionality of pyPheWAS Explorer, the efficacy of the tool in practice should be further tested via a user-centered evaluation, though this is out of scope for the current article.

Despite these limitations, several areas of opportunity exist for the Explorer to enhance EHR analysis. If packaged into a standard application, pyPheWAS Explorer could enable nontechnical clinical experts to easily interact with PheWAS models and identify potentially novel disease associations. Similarly, institutional EHR repositories may benefit from deploying pyPheWAS Explorer as a data exploration and hypothesis generation tool for researchers building EHR analysis cohorts.

## CONCLUSION

pyPheWAS Explorer is a comprehensive tool for exploratory analyses of new EHR datasets. The interactive workflow enables users to quickly answer questions about a dataset’s demographic space and potential for novel phenotypic signatures. We hope that pyPheWAS Explorer’s approachable interface and comprehensive visualizations will empower a broader range of users to delve into the intriguing domain of EHR data.

## Supplementary Material

ooad018_Supplementary_Data

## Data Availability

The data that support the findings of this article’s case study are available from the Synthetic Derivative at Vanderbilt University Medical Center, but restrictions apply to the availability of these data, which were used under license for the current study, and so are not publicly available. An alternative synthetic EHR dataset is available through the pyPheWAS website for testing pyPheWAS and pyPheWAS Explorer (https://github.com/MASILab/pyPheWAS/).
