# Supplementary material for: pyPheWAS Explorer: a visualization tool for exploratory analysis of phenome-disease associations
Source: JAMIA Open. 2023 Apr 3;6(1):ooad018. doi: 10.1093/jamiaopen/ooad018 (PMC10070037; doi:10.1093/jamiaopen/ooad018)
Supplement: ooad018_Supplementary_Data [file ooad018_supplementary_data.zip › pyPheWAS-Explorer_v5_SupplementaryMaterial.docx]

pyPheWAS Explorer: A visualization tool for exploratory analysis of phenome-disease associations

Cailey I. Kerley, Tin Q. Nguyen, Karthik Ramadass, Laurie E. Cutting,

Bennett A. Landman, Matthew Berger

# Supplementary Material

**
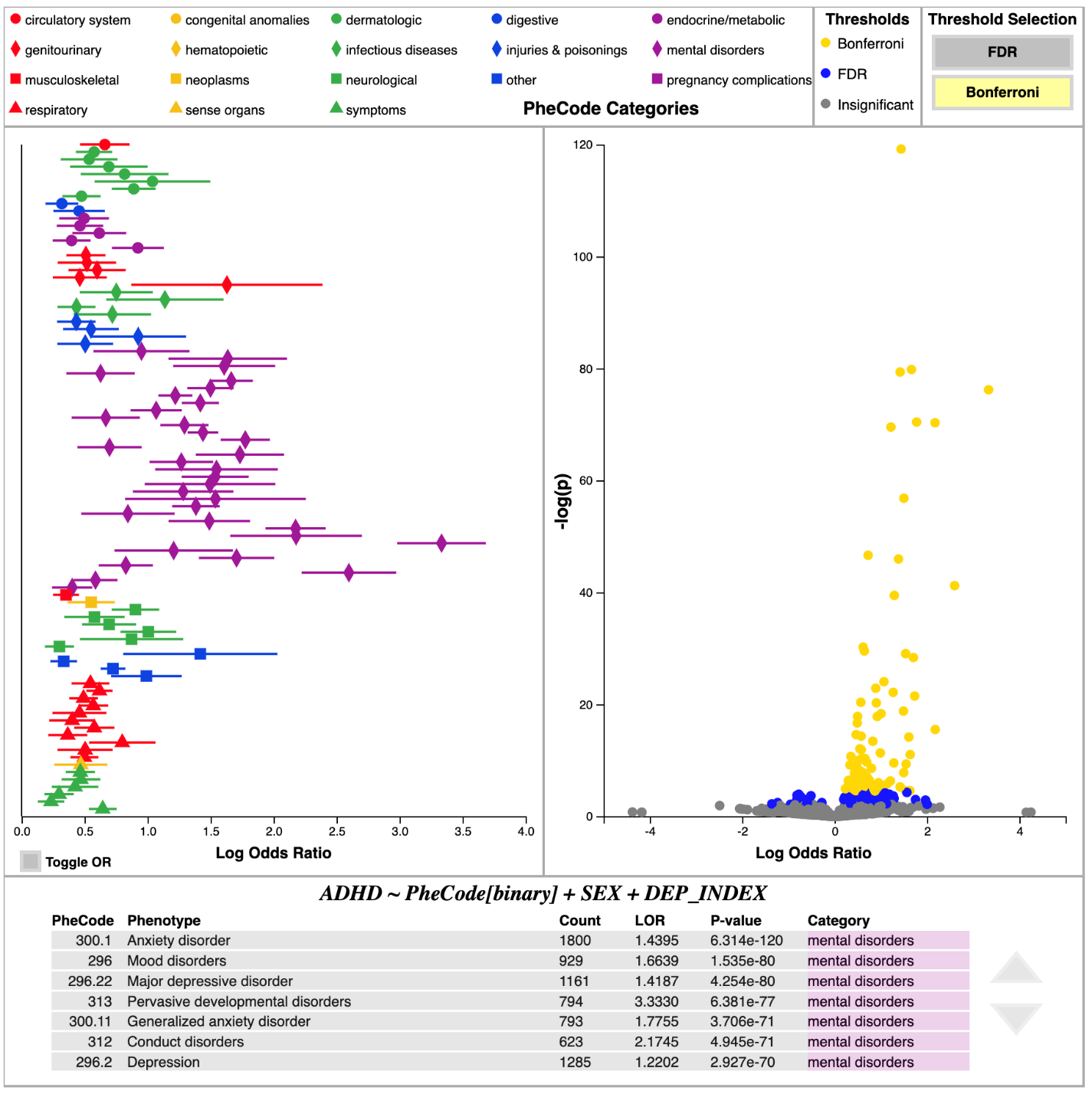
**

**Supplementary Figure 1.** pyPheWAS Explorer Regression Evaluation panel without a selected PheCode. PheWAS results from the binary ADHD model (Figure 2) are shown without any PheCodes highlighted and with Bonferroni multiple comparisons correction applied to the effect size plot. There are many significant ADHD-PheCode associations, a large portion of which fall into the “mental disorders” category. This is clear as well from the data table; the top seven most significant associations are listed, all of which are categorized as “mental disorders”.

**Supplementary Table 1.** Regression results for the three “interesting” PheCode categories from the binary and duration ADHD models. Results include each PheCode-ADHD association’s log odds ratio (Beta) and p-value, along with the PheCode’s category and the number of subjects in the cohort who have a least one instance of that PheCode in their record. All listed associations are significant after Bonferroni multiple comparisons correction. Italicized rows indicate PheCodes with significant ADHD associations in both model types.

| Binary Model | | | | | |
| --- | --- | --- | --- | --- | --- |
| PheCode | **Phenotype** | **Count** | **p-value** | **Beta** | **Category** |
| *706.1* | *Acne* | *655* | *1.32E-23* | *0.890* | *dermatologic* |
| *687.1* | *Rash and other nonspecific skin eruption* | *915* | *4.82E-15* | *0.575* | *dermatologic* |
| 939 | Atopic/contact dermatitis due to other or unspecified | 798 | 7.34E-10 | 0.476 | dermatologic |
| *689* | *Disorder of skin and subcutaneous tissue NOS* | *347* | *3.02E-06* | *0.536* | *dermatologic* |
| 704.1 | Alopecia | 154 | 4.31E-06 | 0.817 | dermatologic |
| 705.8 | Hyperhidrosis | 97 | 9.26E-06 | 1.038 | dermatologic |
| 694.2 | Other dyschromia | 191 | 1.04E-05 | 0.692 | dermatologic |
| *278.4* | *Abnormal weight gain* | *465* | *1.48E-18* | *0.923* | *endocrine/metabolic* |
| 272.13 | Mixed hyperlipidemia | 391 | 1.58E-08 | 0.617 | endocrine/metabolic |
| 278.1 | Obesity | 803 | 2.43E-07 | 0.397 | endocrine/metabolic |
| 272.1 | Hyperlipidemia | 520 | 7.86E-07 | 0.463 | endocrine/metabolic |
| 261.4 | Vitamin D deficiency | 452 | 8.18E-07 | 0.496 | endocrine/metabolic |
| *465* | *Acute upper respiratory infections of multiple or unspecified sites* | *2127* | *6.07E-31* | *0.618* | *respiratory* |
| *476* | *Allergic rhinitis* | *1469* | *4.31E-21* | *0.569* | *respiratory* |
| *512.8* | *Cough* | *1729* | *1.46E-18* | *0.498* | *respiratory* |
| *465.2* | *Acute pharyngitis* | *1639* | *2.11E-17* | *0.491* | *respiratory* |
| *464* | *Acute sinusitis* | *844* | *8.78E-13* | *0.547* | *respiratory* |
| 483 | Acute bronchitis and bronchiolitis | 738 | 1.26E-12 | 0.577 | respiratory |
| *497* | *Bronchitis* | *269* | *2.69E-09* | *0.799* | *respiratory* |
| *495* | *Asthma* | *741* | *3.81E-06* | *0.366* | *respiratory* |
| 512.1 | Wheezing | 360 | 6.44E-06 | 0.504 | respiratory |
| 479 | Other upper respiratory disease | 523 | 1.54E-05 | 0.401 | respiratory |
| 478 | Throat pain | 379 | 2.68E-05 | 0.459 | respiratory |
| Duration Model | | | | | |
| PheCode | **Phenotype** | **Count** | **p-value** | **Beta** | **Category** |
| *706.1* | *Acne* | *655* | *4.33E-14* | *0.248* | *dermatologic* |
| *687.1* | *Rash and other nonspecific skin eruption* | *915* | *1.10E-07* | *0.145* | *dermatologic* |
| *689* | *Disorder of skin and subcutaneous tissue NOS* | *347* | *1.94E-05* | *0.369* | *dermatologic* |
| *278.4* | *Abnormal weight gain* | *465* | *1.65E-07* | *0.248* | *endocrine/metabolic* |
| *465* | *Acute upper respiratory infections of multiple or unspecified sites* | *2127* | *1.01E-13* | *0.071* | *respiratory* |
| *465.2* | *Acute pharyngitis* | *1639* | *1.07E-11* | *0.078* | *respiratory* |
| *476* | *Allergic rhinitis* | *1469* | *1.03E-09* | *0.073* | *respiratory* |
| *512.8* | *Cough* | *1729* | *6.43E-08* | *0.062* | *respiratory* |
| *497* | *Bronchitis* | *269* | *1.12E-06* | *0.549* | *respiratory* |
| *495* | *Asthma* | *741* | *1.51E-06* | *0.057* | *respiratory* |
| *464* | *Acute sinusitis* | *844* | *1.24E-05* | *0.094* | *respiratory* |


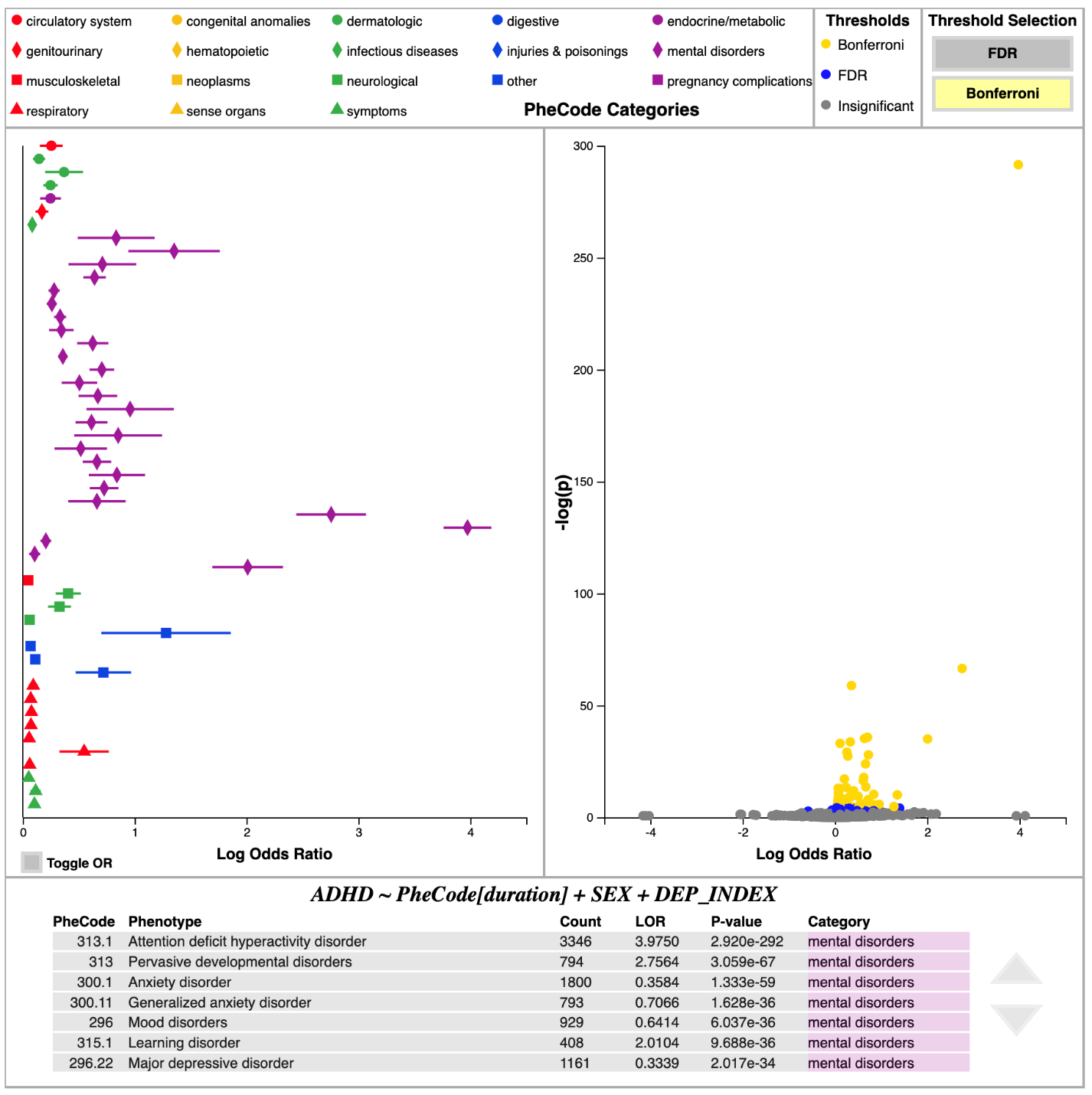


**Supplementary Figure 2.** PheWAS results from the duration ADHD model are shown in the pyPheWAS Explorer Regression Evaluation panel with Bonferroni multiple comparisons correction applied to the effect size plot. Compared to the binary model, there are overall fewer significant PheCode associations. Again, the “mental disorders” category is the most prominent. Many of the PheCode associations from the “interesting” categories in the binary model (“dermatologic”, “endocrine/metabolic”, and “respiratory”) are also significant in this model.
